# THz Sensing With Anomalous Extraordinary Optical Transmission Hole Arrays

**DOI:** 10.3390/s18113848

**Published:** 2018-11-09

**Authors:** Irati Jáuregui-López, Pablo Rodriguez-Ulibarri, Sergei A. Kuznetsov, Nazar A. Nikolaev, Miguel Beruete

**Affiliations:** 1 Antennas Group-TERALAB, Universidad Pública de Navarra, Campus Arrosadía, 31006 Pamplona, Spain; irati.jauregui@unavarra.es (I.J-L.); pablo.rodriguez@unavarra.es (P.R.-U.); 2 Rzhanov Institute of Semiconductor Physics SB RAS, Novosibirsk Branch “TDIAM”, Lavrentiev Ave. 2/1, 630090 Novosibirsk, Russia; SAKuznetsov@nsm.nsu.ru; 3 Novosibirsk State University, Pirogova St. 2, 630090 Novosibirsk, Russia; 4 Institute of Automation and Electrometry SB RAS, Koptyug Ave. 1, 630090 Novosibirsk, Russia; nazar@iae.nsk.su; 5 Institute of Smart Cities Campus Arrosadía, 31006 Pamplona, Spain

**Keywords:** metasurface, sensing, thin film, terahertz, anomalous EOT

## Abstract

Subwavelength hole array (HA) metasurfaces support the so-called extraordinary optical transmission (EOT) resonance that has already been exploited for sensing. In this work, we demonstrate the superior performance of a different resonant regime of HA metasurfaces called anomalous EOT, by doing a thorough numerical and experimental study of its ability in thin-film label-free sensing applications in the terahertz (THz) band. A comprehensive analysis using both the regular and anomalous EOT resonances is done by depositing thin layers of dielectric analyte slabs of different thicknesses on the structures in different scenarios. We carry out a detailed comparison and demonstrate that the best sensing performance is achieved when the structure operates in the anomalous EOT resonance and the analyte is deposited on the non-patterned side of the metasurface, improving by a factor between 2 and 3 the results of the EOT resonance in any of the considered scenarios. This can be explained by the comparatively narrower linewidth of the anomalous EOT resonance. The results presented expand the reach of subwavelength HAs for sensing applications by considering the anomalous EOT regime that is usually overlooked in the literature.

## 1. Introduction

The discovery of extraordinary optical transmission (EOT) through a subwavelength hole array (HA) by Ebbesen et al*.* [1] contributed decisively to relaunch the topic of plasmonics opening new avenues towards the use of apertures much smaller than the operation wavelength [2,3]. Although initially interpreted as the coupling of light to surface plasmons, it was soon noticed that similar peaks could be obtained even with perfect conductors [2,3,4]. This enabled the replica of the phenomenon at frequencies in which metals do not follow a Drude model (typical of the plasmonic approach), such as millimeter-waves [5]. Nowadays, EOT has been found all along the electromagnetic spectrum [2,3] giving rise to disruptive technological applications such as structural color pixels [6,7], metamaterial devices [8,9,10], etc. Interestingly, the high field intensity near the subwavelength apertures at the EOT resonance has been exploited for sensing applications [11,12,13] and nowadays one can find in the literature several examples of EOT biosensors [14], sensors combining nanofluidics and nanoplasmonics [15], and even sensing platforms for a direct detection and monitoring of viruses [16]. There are excellent reviews in the recent literature accounting for the latest progress in this exciting and expanding topic [17,18,19,20]. 

Sensing applications are also gaining momentum in the terahertz (THz) range. This portion of the electromagnetic spectrum goes from 0.1 to 10 THz and is far less developed than the infrared (or microwaves) due to the historical difficulties in generating and detecting radiation at these frequencies. However, a series of breakthrough discoveries made this band accessible, bridging effectively the “THz gap” [21]. Currently, THz spectroscopy and imaging are emerging fields that find applications in a variety of sectors such as security, defense, pharmaceutics, etc. [22,23]. THz spectroscopy is considered to be promising for label-free sensing of substances because this radiation is sensitive to weak molecular interactions, it can deeply penetrate optically opaque materials of non-polar structure and is crucial to detect and identify biological samples, explosives, plastics, semiconductors, superconductors, while having a non-ionizing impact on matter due to a low energy of electromagnetic quanta [24,25]. Nevertheless, a major limitation is the relatively large wavelength that makes THz waves largely myopic when the amount of the substance under test is very small. Metasurfaces (of which EOT HAs are a particular example) are revolutionizing sensing all along the electromagnetic spectrum and especially at THz [24,25,26], because they produce a high electric field intensity near the metasurface, enhancing the light-matter interaction with the substance analyzed and producing a sharp change in the spectral response, usually a shift of the metasurface resonance. This allows for a reliable detection even with minute amounts of analyte, a feature that is optimal for label-free thin-film sensing analysis.

The first example of a thin-film sensor based on EOT HAs operating at THz was reported in [27]. In [28], a thorough study of the sensing performance of a fishnet structure composed of two stacked EOT HAs was evaluated. The sensing capability was assessed in terms of both the amplitude modulation and the frequency shift of the EOT resonance, showing that both strategies could be used for thin-film sensing. Typical EOT sensors consist of HAs with a square unit cell. Nevertheless, as we demonstrated in the past, a rectangular unit cell provides a richer response allowing for the excitation of two different EOT resonances depending on the polarization of the wave, called regular and anomalous EOT [29]. As demonstrated in that paper and analyzed in depth in [30,31], the anomalous EOT resonance is excited when the wave is polarized along the short hole periodicity and the HA is loaded with a dielectric slab with a minimum thickness and permittivity. On the other hand, the regular EOT is the classical EOT resonance that happens for a polarization parallel to the long HA periodicity and can exist even in absence of a dielectric slab. It is worth mentioning that the effect of adding a dielectric layer to an EOT HA had been studied in the past [32,33], but always considering the effect on the regular EOT resonance. Up to now, anomalous EOT has been exploited to develop compact THz polarizers even with dual band operation [34,35], and has been combined with an artificial wire medium for an accurate control of the resonance [36]. In that paper, its potential as a biosensor was pointed out, although it was not realized in practice. Despite the fact that other types of sharp spectral peaks such as Fano resonances have been extensively exploited for metamaterial sensing [37,38,39], these structures usually suffer from frequency shift saturation when the analyte rises a few dozens of micrometers. Interestingly, there is no theoretical saturation in the anomalous EOT resonance frequency. Hence, the only limitation is the attenuation of the peak due to material loss.

In this paper, we perform a thorough analysis of the sensing capability of several HAs loaded with dielectric slabs of different thicknesses, in such a way that some of the structures support the anomalous EOT resonance whereas others are in the limit or do not support it at all. We start the analysis by comparing the features of anomalous and regular EOT in idealized structures, based on purely numerical simulations. Then, we do a thorough analysis of the sensing performance by considering realistic structures in a variety of scenarios. To do this, we deposit thin layers of a dielectric analyte on the structures, and calculate both the sensitivity and the Figure of Merit (FOM, a finer parameter to assess the performance of sensing devices) of each structure. We conclude the study by comparing quantitatively the results obtained both for the regular and anomalous EOT resonance regimes and demonstrate that the optimal operation occurs at the anomalous EOT resonance with deposition on the non-patterned side.

## 2. Materials and Methods

**Figure 1:**
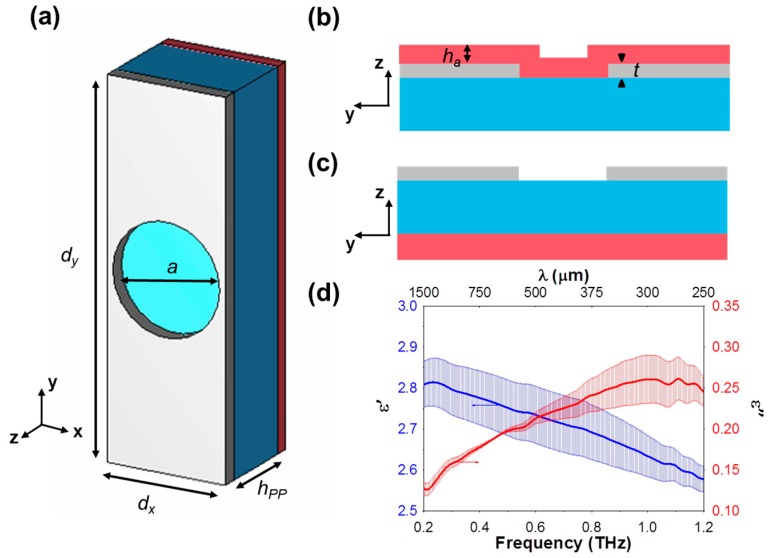
(**a**) Front view and (**b**,**c**) cross-section of the metasurface unit cell, showing the metallization (gray), PP substrate (blue) and analyte (red). Deposition of the analyte is done on the HA (**b**) and PP (**c**) faces. Dimensions: *d_x_* = 115.5 µm, *d_y_* = 350 µm*, a* = 105 µm, *h_PP_* = 50; 75 µm, *t* = 0.5 µm, *h_a_* = 3; 7; 10; 13 µm. (**d**) Measured frequency response of the complex analyte permittivity, with error bars: real (blue, left axis) and imaginary (red, right axis) components.

As shown in the unit cell representation of Figure 1a, the metasurfaces studied in this work consist of a periodic array of circular holes etched on an aluminum (Al) layer of thickness *t* = 0.4 µm laying on polypropylene (PP) slabs of two different thicknesses *h_PP_* = 50 and 75 μm. The PP film from GoodFellow Company [40], whose permittivity is evaluated as *ε_PP_* ≈ 2.25 × (1 − *j*10^−3^) [41,42], was intentionally chosen as a substrate material to minimize the dielectric losses of the metasurfaces. The relevant dimensions of the HA unit cell are *d_x_* = 115.5 µm, *d_y_* = 350 µm and a hole diameter of *a* = 105 µm. In this study the excitation of the HA was done at normal incidence using two different linear polarization states: parallel to the large period of the structure (*d_y_*), which corresponds to the regular EOT resonance excitation [5]; and parallel to the short period of the metasurface (*d_x_*), which, under the appropriate conditions, gives rise to the anomalous EOT resonance [29,30,31].

The sensing performance of the metasurfaces was evaluated by depositing a photoresist material (AR-P 3250 produced by ALLRESIST GmbH [43]) of variable thickness (from 3 μm to 13 μm) on them, either on the PP or the HA side using a standard spin coating deposition technique. The photoresist complex permittivity, plotted in Figure 1d, was extracted experimentally from direct transmission measurements of a 100 μm thick liquid cell. In the initial study presented in the next section, a non-dispersive and lossless analyte with permittivity *ε_a_* = 2.65 was considered. This value was chosen as the mean value of the permittivity in the experimental frequency span.

All the design and numerical results in the paper were obtained using the commercial electromagnetic solver CST Microwave Studio®. To model the HA metasurface as an infinite array, the regime of Floquet ports and unit cell boundary conditions applied to the designed unit cell was employed. The Al-layer was modeled as a non-dispersive medium with conductivity *σ* = 1.5 × 10^7^ S/m whose value, according to our earlier study [42], was found to be reduced versus the nominal conductivity of bulky Al due to inherent surface roughness and granularities of PP films [41]. After the design stage, the structures were fabricated via a standard contact photolithography technique [41,44,45] which was specifically adapted to flexible PP-film substrates, whose industrial production does not allow obtaining a liquid material suitable for posterior film deposition via spin coating. Al-metallization was sputtered onto the PP films by using a vacuum thermal deposition method. Prior to sputtering, the PP substrates were treated with a glow discharge in O_2_ atmosphere to improve adhesion of Al to PP. 

The experimental characterization of the designed HA metasurfaces was done on a custom-made terahertz time-domain spectrometer (THz-TDS) developed in the Laboratory of Information Optics at the Institute of Automation and Electrometry SB RAS (Novosibirsk, Russia). This instrument utilizes a conventional TDS scheme based on a mode-locked Er-fiber laser with a second harmonic generation module (*λ* = 775 nm, *τ* = 130 fs, *P* = 100 mW) and a multi-slit photoconductive antenna iPCA-21-05-1000-800-h (Batop GmbH, Germany) used as an emitter of THz waves, which are further detected via electro-optic sampling [46]. The spectrometer enables the complex transmission measurements within the spectral range of 0.1–2.5 THz with a spectral resolution of 10 GHz and a dynamic range of more than 60 dB (@ 0.85 THz).

## 3. Results and Discussion

Before characterizing the sensing performance of the fabricated HA metasurfaces, we begin the study by analyzing the response of an ideal lossless structure. Thus, we model the metallic parts as perfect electric conductors with zero thickness and all dielectric materials are described only by a non-dispersive real permittivity with values *ε_PP_* = 2.25 and *ε_a_* = 2.65. We consider both vertical and horizontal polarizations in order to excite regular and anomalous EOT, respectively, and ascertain which of the two options offers the best results for sensing purposes. As discussed in [30,31] the appearance of the anomalous EOT depends on the dielectric slab characteristics (*h_PP_* and *ε_PP_*) as well as the large HA periodicity, *d_y_*. More specifically, the anomalous EOT resonance cutoff can be calculated with the auxiliary factor *F* = *h_PP_*√(*ε_PP_ −* 1)/*d_y_*, so that if *F* ≥ 0.25, the anomalous EOT peak will appear. In this initial study we fix the thickness of the PP substrate at *h_PP_* = 78.25 μm so that *F* = 0.25 and hence the anomalous EOT is exactly at cutoff. On the other hand, the regular EOT resonance exists even in absence of a dielectric substrate, so for this study a free-standing structure without PP substrate is considered.

**Figure 2:**
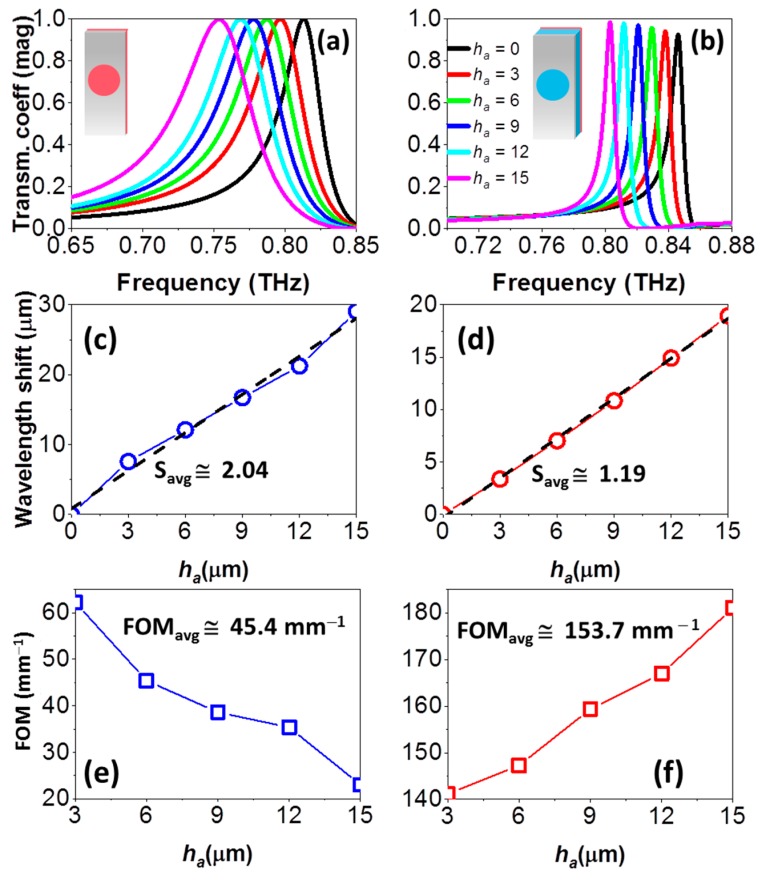
Transmission coefficient for the regular EOT (**a**) and anomalous EOT (**b**) resonance of an ideal lossless HA and several analyte thicknesses (in μm). (**c**) Wavelength shift as a function of the analyte thickness for extremely thin analytes, for the regular EOT resonance, calculated as Δ*λ* = *λ_a_* − *λ*_0_, with *λ_a_* the resonance wavelength at each *h_a_* and *λ*_0_ the resonance wavelength without the analyte. (**d**) Idem for the anomalous EOT. FOM as a function of the analyte thickness, for the regular (**e**) and anomalous EOT (**f**) resonance. Labels S_avg_ in (c), (d) and FOM_avg_ in (e), (f) refer to average values of S and FOM, respectively.

As shown in Figure 2a,b (black line) in absence of analyte, the regular EOT resonance takes place at 0.81 THz, whereas the anomalous EOT resonance occurs at 0.84 THz. To evaluate the performance of each resonance in label-free thin-film sensing applications, a dielectric slab acting as an analyte with permittivity *ε_a_* = 2.65 and thickness ranging from *h_a_* = 3 µm (8.5 × 10^−3^ *λ*_0_, where *λ*_0_~0.35 mm) to 15 µm (42.9 × 10^−3^ *λ*_0_) with a step of 3 µm is added on top. In the anomalous EOT study, the analyte is put on the external face of the PP substrate, whereas, obviously, in the regular EOT case (free-standing) the analyte touches the holey metal. As the analyte thickness increases, the transmission peak redshifts for both resonances, see Figure 2. To have a quantitative assessment of the behavior, the wavelength shift is plotted as a function of *h_a_* in panels (c) and (d). Comparing both plots, it is clear that the shift is stronger for the regular resonance, suggesting at first sight that this regime is more appropriate for sensing purposes. Nevertheless, to clarify this aspect we must carry out a formal evaluation of the performance in terms of the sensitivity (S) and Figure of Merit (FOM), represented in Figure 2e,f. The sensitivity is defined as the ratio between the variation of the resonance wavelength and the analyte thickness, S = ∆*λ*/*h_a_*. With this definition, the average sensitivity is equal to the slope of the regression lines in Figure 2c,d. However, in many cases this value alone is not enough to determine the quality of a sensor. That is why the more refined FOM parameter is usually preferred. The FOM relates the sensitivity and the full width at half minimum (FWHM) in wavelength dimensions, FOM = S/FWHM, and has units of mm^−1^. A sensor with high quality factor would present a narrow spectral line and is able to achieve a relatively high FOM. 

With these definitions, we find that the regular EOT configuration is slightly better than the anomalous EOT in terms of average sensitivity: 2.04 vs. 1.19. However, the FOM shows that the anomalous EOT is clearly superior to the regular EOT resonance, with an average value of 153.7 mm^−1^. This is much higher than the value of 45.4 mm^−1^ calculated for the latter and is due to the comparatively narrower FWHM of the anomalous EOT resonance. With these results, it can be affirmed that the anomalous EOT presents a better behavior for sensing purposes than the regular EOT, improving the FOM by a factor of more than 3.

**Figure 3:**
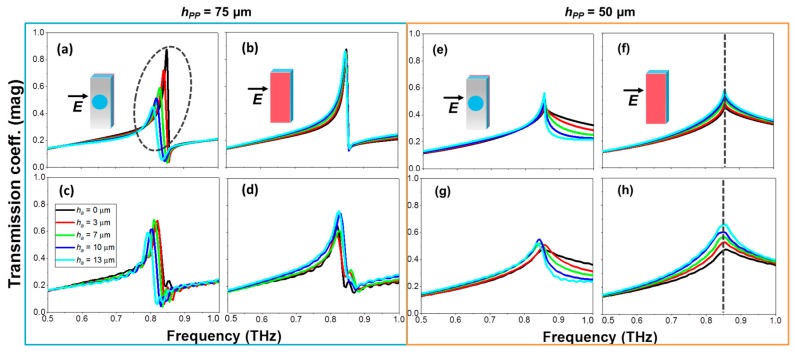
Transmission coefficient magnitude at the anomalous EOT regime in HA metasurfaces with *h_pp_* = 75 µm (**a**–**d**) and *h_pp_* = 50 µm (**e**–**f**) µm under normal incidence and different analyte thicknesses: *h_a_* = 0 µm (black); *h_a_* = 3 µm (red); 7 µm (green); 10 µm (dark blue); 13 µm (cyan) for the anomalous EOT. Simulated (top) and measured (bottom) results. The dashed ellipse in panel (**a**) highlights the region where sensing based on frequency shift is feasible. The dashed grey lines in panels (**f**,**h**) highlights the resonance frequency (0.85 THz) where the amplitude sensitivity analysis can be done. Insets in panels (**a**,**b**,**e**,**f**) depict schematically each scenario, following the colour convention of Figure 1.

**Figure 4:**
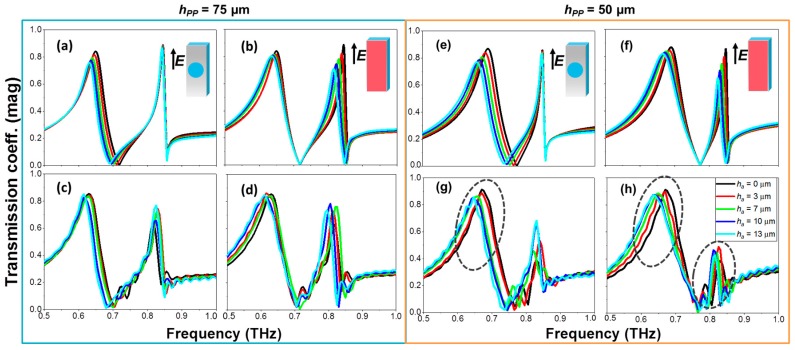
Transmission coefficient magnitude at the regular EOT regime in HA metasurfaces with *h_pp_* = 75 µm (**a**–**d**) and *h_pp_* = 50 µm (**e**–**f**) µm under normal incidence and different analyte thicknesses: *h_a_* = 0 µm (black); *h_a_* = 3 µm (red); 7 µm (green); 10 µm (dark blue); 13 µm (cyan) for the anomalous EOT. Simulated (top) and measured (bottom) results. The dashed ellipse in panels (**f**,**g**) highlights the regions where sensing is feasible. Insets in panels (**a**,**b**,**e**,**f**) depict schematically each scenario, following the colour convention of Figure 1.

After this initial study, we concentrate now on the analysis of the designed and fabricated HA metasurfaces. As our aim here is to evaluate in depth the performance of the anomalous EOT resonance for sensing applications, two different substrate thicknesses are used *h_PP_* = 75 μm (Figure 3) and *h_PP_* = 50 μm (Figure 4), that correspond to *F* = 0.24 and 0.16, respectively. The first case is chosen to have the anomalous resonance very near cutoff, so that a slight change provoked by an analyte can give rise to a strong spectral variation. Conversely, in the second case the anomalous EOT resonance is deeply in cutoff and we do not expect a sharp response, at least with thin analytes. The sensing performance of the structures is evaluated by depositing four different analyte thicknesses: *h_a_* = 3 µm (8.5 × 10^−3^ *λ*_0_,); 7 µm (19.8 × 10^−3^ *λ*_0_); 10 µm (28.3 × 10^−3^ *λ*_0_); and 13 µm (36.8 × 10^−3^ *λ*_0_). Numerical results are shown in the upper panels (a), (b), (e), (f); and experimental measurements on the lower panels (c), (d), (g), (h) of Figure 3 and Figure 4. To have a complete picture of the performance, two different scenarios were considered: when the analyte is deposited on the HA side and on the PP side, schematically depicted in Figure 1b,c. 

Focusing first on the horizontal polarization (anomalous EOT), we find that the transmission coefficient without analyte (*h_a_* = 0) shows in all considered cases clear resonant features at ~0.85 THz, with very good concordance between simulation and measurement, see black curves in Figure 3. Although in the case of the 75 μm thick PP film (Figure 3a–d) the anomalous EOT resonance is slightly below cutoff, it is close enough so that it gives rise to a high transmission peak followed by a minimum in the spectrum. This is in contrast with the behavior of the 50 μm thick PP film (Figure 3e–h) that shows only a local maximum (a “kink”) with reduced amplitude (~0.5), as expected [29,30,31]. Now, the sensing performance of each configuration is analyzed by increasing the analyte thickness. It can be seen that the best scenario for sensing purposes is the one in which the analyte is deposited on the PP side of the *h_PP_* = 75 µm thick metasurface (Figure 3a,c), with an average sensitivity of ~0.8 (~1.24) and an average FOM of ~28.6 mm^−1^ (~46 mm^−1^) in the experimental (numerical) results. This is in agreement with our analysis above, since depositing on the PP side is equivalent to increasing the substrate thickness (as a side comment, note that the peak amplitude decreases as *h_a_* increases due to the growing ohmic loss because, unlike the previous study, we are considering here a lossy and dispersive analyte. Note also that this effect is more evident in the simulation than in the experimental results, probably because in the experiment the characteristics of the analyte might differ between successive depositions and, in addition, it is rather difficult to have a proper characterization of metal and dielectric losses). When the analyte is deposited on the HA side (Figure 3b,d) the frequency shift of the anomalous EOT resonance is negligible, rendering this configuration ineffective for sensing purposes. As explained in our previous paper [31], two different anomalous EOT peaks can be excited independently by placing dielectric slabs on both sides of the holey metal. In the configuration considered here, the analyte slab is too thin and hence unable to excite its own anomalous EOT resonance (i.e., *F* << 0.25 in that side). This is why in the spectral response we only see the peak corresponding to the PP slab, which is largely insensitive to the analyte deposition on the other face.

For the 50 µm PP thick structure with the analyte deposited on the PP side we find that the “kink” becomes narrower and its amplitude grows faintly as *h_a_* is enlarged, see Figure 3e,g. This is because we are approaching gradually towards, but never reaching cutoff, even with the largest analyte thickness. In practice, this means that it might be feasible to perform sensing by looking at the peak amplitude variation. At least in simulation (Figure 3e) this looks viable, but it seems hardly attainable experimentally (Figure 3g), probably due to fabrication tolerances, and to the fact that high Q resonances are greatly affected by losses. When the analyte is placed on the HA side (Figure 3f,h) we notice a negligible frequency shift but, interestingly, a clear amplitude increment of the “kink”. This enhancement in the transmission coefficient is associated with a better impedance matching of the structure. From the specialized literature [47], it is known that the optimal operation of frequency selective surfaces and spatial filters is achieved when both faces of the metallic film are coated with dielectric slabs of identical characteristics. In our case, increasing the analyte thickness leads to a better matching of the impedance seen at both interfaces, giving as a result a higher peak amplitude. In this way, it is possible to define a new sensitivity, referred to as Amplitude Sensitivity (AS) and calculated as the ratio between the variation of the amplitude at the resonant frequency and the variation of the analyte thickness: AS = ∆*A*/*h_a_*. With this definition, we experimentally obtain AS = 0.02 µm^−1^. Note that in this case it is impossible to define a FOM, due to the inexistence of a valid FWHM. If we define the amplitude sensitivity as done in [28], AS_%_ = ∆*A*(%)/*h_a_*, we achieve a maximum experimental value for the case of *h_a_* = 3.2 µm of 35%/µm. Although this value is much lower than the 66%/µm reported in [28], note that in our case it is only needed to tune the spectrum at a single frequency. In addition, our amplitude modulation is done “positively”, and does not experience vanishing of the signal as the analyte increases. Indeed, our device exhibits an amplitude rise as we add the analyte material, because when the analyte thickness is increased, the *F* factor gets closer to the limit condition in which the anomalous peak appears. Obviously, a saturation of the response will arise when we get the condition *F* = 0.25.

We consider next vertical polarization (regular EOT) just for comparison purposes; see all results in Figure 4. In this case, the response is very similar regardless the PP thickness, as this parameter is not critical for the performance (in contrast to anomalous EOT). Therefore, we will study both cases, *h_pp_* = 50 and 75 μm, in parallel. The first difference we observe in the spectral response in comparison with the previous study is that there are two resonance peaks, at 690 and 847 GHz. Each peak is related to the EOT resonance principally at the PP and air interfaces [48]. Therefore, depositing the analyte on the PP side mainly shifts the lower frequency peak whereas depositing on the air side mainly affects the higher frequency one. Although in the simulation both peaks can be potentially employed for sensing purposes, in the measurement only the lower frequency resonance presents a noticeable redshift. Furthermore, the structure with *h_pp_* = 50 µm has a better performance in practice, probably due to the thinner substrate, which is further from the saturation point of the maximally achievable frequency shift. Consequently, we only select the cases highlighted with a dotted ellipse for the calculation of the sensitivity and FOM as these are the ones in which we can appreciate a frequency shift large enough to use the structure as a sensing device, and we have a good agreement between the simulated and measured results. Note that in the case of the second resonance when depositing on the HA side, the FOM cannot be calculated due to a low magnitude of the peak.

To ease the comparison all the values of the cases of interest extracted from the experimental measurements are collected in Table 1. As shown there, although the sensitivity in the anomalous EOT case is below the regular EOT case, the FOM is higher, corroborating our initial study. 

As a final study, we compare our structure with others found in the literature that also exploit sharp peaks in transmission, such as Fano resonances. For this comparison, we use the sensitivity as defined before, S = ∆*λ*/*h_a_*. In [37], a dual flexible THz asymmetric split ring resonator (TASR) was used to detect a thin film of 100 nm with refractive index *n* = 4, by coating both sides of the structure, and achieved an experimental sensitivity of 67 and 85 for the analyte deposited in the non-patterned and patterned side, respectively. In [38], a toroidal TASR was designed with a sensitivity of 3.74 for a 13.8 μm thick analyte with *n* = 1.6. Finally, a TASR based on a Fano resonance operating at 0.52 THz was designed in [39]. The sensitivity achieved for a 1 μm thick analyte of *n* = 1.6 was 11.3. As seen in this comparison, our designs present lower sensitivity values. Nevertheless, the sharp peak of the EOT resonance leads to higher values of FOM. Moreover, the use of the EOT resonance instead of Fano resonances brings the advantage of not having any theoretical limit in the achievable frequency shift.

## 4. Conclusions

To sum up, we have demonstrated the superior performance of a HA metasurface when it operates at the anomalous EOT resonance, exceeding largely the results obtained at the regular EOT in label-free thin-film sensing applications. Although the frequency shift and hence the sensitivity of the anomalous EOT resonance are smaller than those of the regular EOT resonance, its comparatively narrower FWHM leads to an increment of the FOM. In our initial study considering idealized structures we have achieved an average FOM of 153.7 mm^−1^, which improves the results obtained with the regular EOT by a factor of more than 3. Two HA metasurfaces of different PP thicknesses have been fabricated and measured to analyze the effects on the sensing quality parameters depending on the side on which the analyte under measurement is deposited. We have demonstrated that, for sufficiently thick substrates, sensing in the anomalous EOT resonance and depositing on the non-patterned side of the metasurface is a much better option with lower sensitivities but higher FOMs, with an improvement of a factor between 2 and 3 as compared to the best case of the regular EOT resonance. Using the optimal configuration provides a benefit that in routine operation the structure can be cleaned without damaging the metallic pattern. Additionally, we have found an alternative for thin-film sensing based on a variation of a peak amplitude. This can be used when the substrate thickness is too thin to exhibit the anomalous EOT resonance and takes place when the analyte is deposited on the patterned side of the metasurface. The obtained results demonstrate the excellent performance of the anomalous EOT resonance in practical thin-film sensing platforms.

## Figures and Tables

**Table 1:** Average sensitivity and FOM achieved in the configurations shown in Figure 3 and Figure 4, experimental results.

Resonance	*h_pp_* (μm)	Analyte Side	S	FOM (mm^−1^)
Anomalous	75	PP	0.8	28.6
		HA	−	−
	50	PP	−	−
		HA	0.02 µm^−1^ *	−
Regular	75	PP	−	−
		HA	−	−
	50	PP	1.2	12.9
		HA	1.85/0.68 **	16.6/− **

* Note that in these cases we are referring to the amplitude sensitivity, AS = ∆*A*/*h_a_*. ** The first/second number refers to the first/second resonance
